# Elements of Materia Medica and Therapeutics

**Published:** 1846-01

**Authors:** 


### Elements of Materia Medica and Therapeutics. By *Edward Ballard*, M.D. Lond. and *Alfred Garrod*, M.D. Lond. 8vo. pp. xxiv—447. London: Taylor and Walton, 1845.

It is not surprising, when we consider the nature and variety of the subjects treated of in works on Materia Medica that they should be less frequently than almost any others brought under our notice. Independent of the acquirements requisite for the preparation of the more extended and complete works of this class, their necessary size and expence, must limit the number, inasmuch as a remunerating sale cannot be expected for more than a few, and those of the highest authority. The invaluable Cyclopædia, misnamed "*Elements* of Materia Medica and Therapeutics," by Dr. Pereira, and the excellent systematic works of Drs. Thomson and Christison, and the Dictionary of Brande, have acquired a celebrity and authority, at once so extended and so just, as to defy much competition. Of smaller manuals and compendiums, for the most part compiled from the larger works above-mentioned, there are many, and some excellent of their kind. It is, however, remarkable, that hitherto, no work of an intermediate character should have emanated from the British press. We make no allusion to the valuable and erudite work of the accomplished President of the College of Physicians, because the Pharmacologia of Dr. Paris must be considered a work sui generis, altogether different in its aim and character (more particularly the last edition) from those of ordinary treatises on Materia Medica and Therapeutics.

"In introducing," say the authors of the present volume, "another work upon Materia Medica, in addition to those already before the public, the writers desire that it should be looked upon as strictly elementary; and, in so far as the description of the drugs is concerned, nothing more than a compilation. The necessity of this outline has been pressed upon them for some time by students with whom they are respectively connected, as well as by gentlemen in considerable practice, both of whom felt the inconvenience of reading through extended treatises, with a view to those essential points of instruction which might be conveyed in fewer words and be less incumbered with extrinsic matter. Without depreciating, then, the larger works in our language as books of reference, the present is intended to be one every word of which the student ought to read, and with whose entire contents he should render himself familiar."—*Preface.*

This account of the character and scope of their work, as given by the authors themselves, might exonerate us from the duty of an extended critical examination of the individual subjects of which they treat, on the assumption that, as stated in their preface, they have "carefully collated" and "consulted" "the larger works of Drs. Thomson, Pereira, and Christison, and the established authors on Chemistry, Natural History, and Medicine." Nor is it our intention, in what follows, to attempt more than to offer a few remarks on the manner in which the authors have performed their task, and give our readers such an account of the volume before us as may enable them to judge how far it supplies, what we believe, would be considered a deficiency in medical literature.

Keeping in view, therefore, the design of the work, and the necessity for economising space as well as matter, we cannot but object to the occupation of no less than thirty pages with a table of contents, which is altogether useless for the purposes of reference, while the index, which, in all works of this kind, should be as full and complete as possible, occupies but two pages and a half, and is sadly defective. The volume very properly commences with a therapeutical introduction by Dr. Ballard, which occupies thirty pages. Judiciously avoiding any attempt to define the actual modus operandi of medicinal agents, and to classify them accordingly, Dr. Ballard contents himself by stating certain general principles, which have been ascertained chiefly through the medium of modern chemistry. With reference to the chemical composition of the active principles of medicines, he alludes to the singular fact, " that in several of those, whose operation upon the nervous system is manifested in the most energetic manner, a certain similarity prevails," not only as regards the relative proportion of their carbon, hydrogen, and oxygen, but also in the existence of a minute proportion of nitrogen, as indicated in the formulæ of the following alkaloids :—

| | | | |
|---|---|---|---|
| Solania | $C_{84}H_{68}N\,O_{28}$ | Strychnia | $C_{44}H_{23}N_2O_4$ |
| Morphia | $C_{35}H_{20}N\,O_6$ | Brucia | $C_{44}H_{25}N_2O_7$ |
| Codeia | $C_{35}H_{20}N\,O_5$ | Quina | $C_{20}H_{12}N\,O_2$ |
| | | Cinchonia | $C_{20}H_{12}N\,O$ |

It would appear, therefore, that when, on analysis, any vegetable drug is found to possess so remarkable a composition as this, we have good grounds for predicating its activity.

It is observed also, that other elementary substances derived from the inorganic kingdom, and which in their chemical characters are allied to nitrogen, remarkably influence the functions of the nervous system, *e. g.* antimony, arsenic, and phosphorus ; and it is hinted that the same analogy probably exists between the operation and chemical relations of other natural groups of elementary bodies.

As regards the general mode in which medicines influence the functions of the body, Dr. Ballard adopts the views of Professor Liebig. Putting out of consideration those agents which exert a *directly* mechanical or chemical influence, the effects of medicines may be said to be of two kinds, either an increase or diminution of the energy of the vital functions, as displayed in the phenomena of sensation or motion ; or else a material alteration in the formation and composition of the secretions eliminated from the blood. In reference to these two classes of effects, Dr. B. says :

" 1st. The perception of a sensory impression, and the origination of a motor influence, depend upon the mode in which the cerebro-spinal centres perform their allotted functions, and are accompanied by a transformation of their tissue. Medicines which influence either of these, therefore, might be supposed to act primarily upon the nervous system, and to modify in some way the chemical metamorphoses which it undergoes. The active principles of *Opium, Nux Vomica, Cinchona Bark* and *Ipecacuan,* for example, might be ' supposed to take a share in the formation of new, or the transformation of old brain and nervous matter.' When, therefore, we enquire how far these active principles, which diminish sensibility, produce tetanic spasm, increase muscular tone, or diminish tone and induce vomiting, are fitted by their composition to act in the manner indicated, the singular result announced by Professor Liebig, is obtained, ' that the composition of the most active remedies, viz. the vegetable alkaloids, cannot be shewn to be related to that of any constituent of the body, except only the substance of the nerves and brain.' They all contain a minute amount of nitrogen, very much less than is discovered in the compounds of *Proteine,* approaching in this respect, the fats which contain none. ' If it must be admitted as an undeniable truth,' argues this distinguished authority, ' that the substance of the brain and nerves is produced from the elements of vegetable albumen, fibrin or caseine, either alone, or with the aid of the elements of non-azotised food, or of the fat formed from the latter ; there is nothing absurd in the opinion, that other constituents of vegetables, intermediate in composition between the fats and the compounds of proteine, may be applied in the organism to the same purpose."

" 2nd. In considering the mode in which remedies of this class alter the quantity of a secretion, or modify its chemical composition, two principal circumstances must be kept in view. It must be recollected that the secreting organs are merely the agents by which the material is separated from the blood, and the amount of the secretion will depend on the circulation through them, and the healthy state of their nervous supply. The separated matters themselves, however, do not originate here : they are derived from the transformations of tissue, which are constantly proceeding, with greater or less rapidity, in the body at large. Medicines, therefore, which affect secretions, must do so in one of two ways; either they must act upon the nervous centres, increasing or diminishing their influence upon the organs, and modifying the circulation through them, or, in the words of the chemist before quoted, 'they must take a direct share in the change of matter in the body,' or 'exert an influence on the formation, or on the quality of a secretion by the addition of their elements.' " P. 6.

This statement of Liebig's views of the operation of medicines, is, perhaps, sufficiently " plain to him that understandeth," but it may be questioned whether the generality of the readers for whom this volume is chiefly intended, will derive from it that clear view of the subject which its importance demands. For, that the subject is one of the highest interest and importance cannot be denied; and though we may not as yet be in a condition to make any very satisfactory practical application of these doctrines in the selection and use of individual medicines, we have no hesitation in asserting our belief that, organic chemistry has pointed out to us the path to pursue in our therapeutic enquiries. The chemical evidence which has been adduced of the specific influence exerted by remedial agents on particular tissues, by modifying the healthy processes going forward in them, in virtue of certain chemical relations existing between the tissues and the products from them on the one hand, and the medicinal agent on the other, derives strong confirmation, both from recent experiments on the action of poisons and from clinical observation. We have, it is to be feared, been in the habit of confining our attention far too much to the more immediate and ostensible effects of our remedies; and whilst our treatises on Materia Medica and Therapeutics are full of references to the purgative, emetic, stimulant, sedative, and other of the more rude and manifest operations of medicines, there has been too great a disposition to deny, or discountenance, all statements with reference to the specific influence of particular remedies on definite forms of morbid action. However much there may be of fiction and delusion in the statements of the Homœopathists concerning the specific adaptation of their infinitessimal doses of particular medicines to forms of disease denoted by special symptoms, we are inclined to believe that legitimate medicine, may, in this respect, as well as in some others, have cause to admit that the author of the " Organon of Rational Medicine" has done good service to the healing art. Amidst all the vagaries and delusions of Homœopathy, Hydropathy, and even Mesmerism, we must not forget that " fas est, et ab hoste doceri." Undoubtedly many of those medicines whose therapeutic virtues are most to be depended on in the diseases for which they are chiefly administered, are of the class which afford the strongest confirmation of the views which refer their curative agency to their adaptations to modify the molecular changes going forward in the tissues and organs assumed to be the seat of disease.

The characters and operation of the various classes of medicinal agents are briefly discussed under the ordinary heads of Tonics, Stimulants, &c. and although there are here not a few statements to which exception might be taken, the remarks are, for the most part, useful and judicious. We think, however, that the conditions of system calling for the use of medicines of a particular class and general rules for their exhibition, should have been stated more definitely, and at greater length. Under the head of Narcotics, for example, the remarks are so very general as well as brief, as to fail of giving the student the requisite assistance in their therapeutic employment. But, to enable our readers to judge for themselves of the character of this part of the volume, we extract the entire section, headed "*Narcotics.*"

"*Narcotics* are remedies which lessen the manifestation of vital phenomena dependant upon the nervous system, especially deadening sensibility and diminishing the motor power, more, however, by reducing the energy of voluntary effort than by paralysing the parts concerned. Their full operation is sleep or coma. *Opium* is the type from which most descriptions of the class have been drawn; but although many narcotics agree with it more or less, yet there are peculiarities to be remarked in the action of each. It is generally supposed that they are primarily stimulant; but this part of their action is very trifling as these medicines are ordinarily prescribed. So great a stress, however, does Dr. A. T. Thomson lay upon it, that he attributes the sedative effect to the consequent collapse. 'Narcotics,' he says, 'strictly so called, operate as diffusible excitants; and this so decidedly, that, by regulating the doses and the repetition of them, the sleep which generally follows their administration may be altogether prevented, and the exciting influence of the medicine only be obtained: now, the effect of a direct sedative is an immediate depression of the powers of the system without any apparent previous excitement. The symptoms of collapse which follow those of excitement, after the administration of a narcotic, are the consequence of the excitement.' This is an opinion by no means generally held; and though, in some instances, the stimulant operation cannot be denied, yet we are rather disposed to regard the two actions as distinct, than as consequent the one upon the other. It would be foreign to our purpose to enter at any length upon the manner in which narcotics manifest their action upon the several functions of the body, differing as they do in this respect among themselves. Suffice it to say, that, as a part of their general sedative influence upon the nervous system, they diminish the secretions of the liver and kidneys, arrest more or less the performance of those functions which severally attach to the different parts of the alimentary canal, retard digestion and constipate the bowels, both by lessening the secretions poured into them and rendering their movements sluggish. The difference of operation of the several members of the class will be most conveniently considered when they come under special review. All we will observe at present is, that some dilate, while others contract the pupil; some appear to concentrate their sedative action more particularly upon the functions of the encephalon, others upon the contractile power of the alimentary or bronchial tubes, while a strict distinction is to be drawn between those which occasion constipation and those which do not; all these things being of great practical importance. As in the case of the two former classes, we are of opinion that the primary action of narcotics is exerted upon the nervous centres, but concentrated most especially upon the brain.

"The objects which are mostly in view in the administration of narcotics, are the production of sleep, or the alleviation of pain and spasm. The former is sometimes a most important point to be attained; and, though pain is frequently nothing more than the effect of increased vascularity in a part, it is, nevertheless, a symptom for which the practitioner is often specially required to prescribe, and where there is nothing of this nature to occasion it, the use of narcotics is particularly indicated. Their antispasmodic value rests upon the same ground with their utility as anodynes." P. 12—14.

The therapeutical is followed by a chemical introduction by Dr. Garrod, which, however, is an introduction only to organic chemistry. For this Dr. G. apologises by stating that—

"It does not come within the limits of a work like the present, to enter at large into chemical minutiæ; and we must therefore pre-suppose in our readers a general knowledge of the principles at least of inorganic chemistry; and the object of the present introduction will be to give a concise statement of the principles of the organic branch of the science, and to furnish a connected view of the composition and leading properties of the compounds contained in the vegetable department of the Materia Medica." P. 32.

Why a general knowledge of the principles of organic as well as of inorganic chemistry should not be presupposed in those who are studying Materia Medica, we are at a loss to understand. An apology was more needed for the introduction of the one than for the omission of the other. Both are altogether out of place in any work on Materia Medica and Therapeutics, and more especially in one of the present character, professing to confine itself to the leading facts and doctrines of the science. Besides, "the composition and leading chemical properties of the compounds contained in the vegetable department of the Materia Medica," are necessarily given under their respective heads. However well qualified, therefore, the author of this introduction may be (as we willingly admit that he has shewn himself) to give a good summary of the science of organic chemistry, which may be highly useful to both student and practitioner, its introduction into the body of a manual of Materia Medica can be accounted for only on the supposition that the author has allowed his better judgment to be blinded by his love for a favorite science.

None but those articles which are rendered officinal by the London College being admitted into the body of the work, the inorganic Materia Medica is comprised in 90 pages; the articles being arranged according to their chemical relations. We have been much surprised to find that the proportions of the various articles entering into the composition of the Galenical preparations, are almost invariably, and evidently by design, omitted. This is undoubtedly a signal defect, of which we fancy the young gentlemen preparing for Apothecaries' Hall, if none others, will have just cause to complain. Under the head of Arsenic, for example, the following is all that is said of—

"*Liq. Potassæ Arsenitis.*—(Arsenious Acid and Carbonate of Potash, dissolved in water, and coloured with compound tincture of lavender.) It is more fitted for internal use than the solid acid, as it may be much diluted and rendered thus less irritating to the stomach." P. 126.

Now as this preparation is incorrectly designated in the London Pharmacopœia, and as the excess of alkali very materially modifies its chemical relations, a knowledge of the proportions of arsenious acid and carbonate of potash entering into its composition becomes important.

There is a similar omission of the synonyms of the medicines, with many of which it is absolutely necessary for every medical man to be acquainted. Even of those that are in common and daily use, there are not a few of which the student may fairly be supposed to be ignorant, and expect to be informed of, in such a work as this, especially those synonyms which have been but recently replaced by the present names.

Each article is described under the following heads: Physical Properties, Preparation, Chemical Composition, Chemical Relations, Operation and Uses, Dose and Officinal Preparations. The chemical composition and relations are given for the most part, though very succinctly, with great clearness, and illustrated frequently by diagrams. In many instances, however, both the composition and the special therapeutic adaptations of the officinal preparations are very imperfectly stated. As an illustration we subjoin the—

"*Mist. Ferri Composita.*—(Myrrh, carbonate of potash, sulphate of iron, and spirit of nutmeg, sugar, and rose-water.) The alkaline carbonate re-acting on the sulphate of iron, gives rise to the carbonate of iron in the mixture. It is a very valuable preparation; its slightly stimulant properties rendering it highly useful in some cases of anæmia and in amenorrhœa. Its constipating effect may be obviated, by the combination of some decoction or extract of aloes with it, while its stimulating action upon the pelvic viscera is augmented." P. 121.

Now, from this, the student would certainly not gather either the chemical, or therapeutic peculiarities, of this very useful preparation. Some reference should undoubtedly have been made to the important part played by the sugar and the excess of alkali, both as regards the chemical constitution and therapeutic virtues. Distinct reference should also have been made to the changes which the mixture undergoes by exposure to the air.

The vegetable Materia Medica is preceded by a brief sketch of the natural classication of plants, in which are given the principles of botanical classification, the leading characters of the classes, sub-classes, and orders, with illustrative diagrams, on the plan adopted by Professor Lindley in his School Botany, and intended to aid the student in comprehending the essential structure of the flower in each natural class. With reference to this Botanical Introduction, (as well as to a similar one, preceding the Animal Materia Medica, in which a sketch of the classification of the animal kingdom is given,) the same remarks apply, which we have felt called to make, when speaking of the chemical part. And certainly if it be desirable to give (as perhaps it may) the essential botanical characters of the various orders of plants, they would have been far more naturally and usefully inserted, after the headings, under which the substances derived from each class are given. Of the diagrams we cannot avoid observing that, if intended for those only who have learnt their botany from Dr. Lindley and his writings, they were not needed; and, if for others, some farther explanation is required, than what is afforded by the footnote, in order to render them available for the purpose for which they were intended. Of the meaning of the algebraic symbols given to illustrate the characters of the flowers in the two grand divisions of Exogens and Endogens, we are fain to confess our entire ignorance. We will venture to say, that nineteen-twentieths of our readers will be greatly puzzled to know what is meant by "Flowers $^{4\,5}\surd$" or "Flowers $^{3}\surd$" We object not to the employment of such symbols, which may be very convenient and useful, but as they are purely arbitrary, every one who employs them is bound to tell us what they mean.

We had noticed various inaccuracies in reference to both the commercial and botanical sources of some drugs, but our space warns us not to attempt any detailed criticism of this kind. We may mention, however, that East Indian Kino is not obtained, as here stated, from the Nauclea Gambir, but from a species of Pterocarpus; not P. Erinaceus, as was at one time supposed, but P. Marsupium. All the varieties of Catechu are spoken of, as derived from Acacia Catechu, whereas, it is well known, that they are obtained from at least three distinct plants—the Areca Catechu, and Uncaria Gambir, as well as the Acacia Catechu.

Again, under the head of Cinchona scarcely any attempt appears to have been made to refer the various commercial articles to their right sources. Aconite is referred to the species napellus, but as the London Pharmacopœia (though perhaps without any sufficient grounds) refers it to paniculatum, this ought at least to have been mentioned, and accompanied with the statement that it is scarcely ever to be met with in the British market. Where, as in the case of Hemlock, the plant is frequently confounded and mixed with others, some notice of these should have been given. We subjoin a specimen of this part of the work.

VALERIANA (*Radix*).

" DESCRIPTION.—*Form*, a short tuberose rhizome, from which radical fibres come off, three or four inches in length, which are the officinal part. *Colour*, yellowish-brown when dry. *Odour*, fætid and peculiar, and not disagreeable in the fresh plant. *Taste*, warm, camphoraceous, and nauseous.

" CHEM. COMP.—Its most important ingredients are, *volatile oil*, *valerianic acid*, *resinous* and *gummy* matters.

" The *volatile oil* is obtained by distillation of the root with magnesia, in order to fix the valerianic acid. It is of a light-green colour, and lighter than water, having the odour of the valerian. See Appendix.

" The *valerianic acid*, when set free from the magnesia by sulphuric acid, occurs as an oily liquid, having an odour similar to the oil, but an acrid acid taste. It is probably formed by oxidation of the oil. It can be procured artificially by oxidation of the *oil of potatoes* or *grain spirit*, to which it stands in the same relation as acetic acid does to alcohol. It forms soluble salts, with bases, as oxide of zinc and quinine, which have lately been introduced into medicine. *Formula*, $HO+C_{10}H_9O_3$.

" OPER. AND USES.—Valerian is stimulant and antispasmodic. We have found it of some value in removing the *paroxysms of headache*, which occur in atonic dyspepsia anæmia. It has obtained a reputation in convulsive affections, as *epilepsy*, *hysteria*, and *chorea*. We have seen it of service in hysteria, but never in epliepsy.

" DOSE.—℈j—ij.

" OFF. PREPS.—*Infusum Valerianæ*. (Valerian macerated in boiling distilled water, and strained). *Dose*, f ℥j—ij.

" *Tinctura Valerianæ*. (Bruised valerian macerated in proof spirit, and strained.) *Dose*, f ʒj—iv.

" *Tinctura Valerianæ Composita*. (Bruised valerian macerated in aromatic spirit of ammonia, and strained.) *Dose*, f ʒss—ij. More stimulant than the two former preparations." P. 265.

In the remarks which we have been called to make, we have had no wish to detract from the real merits and utility of the volume before us. In proportion, however, as the knowledge, which a work like the present is intended to impart, is condensed, it ought to be accurate, and in proportion as the limits allowed are restricted, they should be occupied with the proper and legitimate objects of materia medica. If, instead of introducing subjects which do not belong to the science, the authors had devoted more space to the characters and medicinal virtues of the different substances and to the special indications they are calculated to fulfil, the volume would have been more useful to both the medical student and practitioner, and might have claimed a higher character. The merited reputation of the authors, led us, perhaps, to form higher expectations of their work than we ought, or than have been realized. An unfavourable impression, as it regards the care and attention which have been bestowed in its compilation, is necessarily produced by finding, at the end of the volume, a "list of preparations omitted," and this impression is not removed by such faults of style as the following. "Purgatives are medicines which, within a short, or given time, after exhibition, produce the evacuation of the *bowels*, whether *they* have been received through the stomach or applied more immediately to the rectum."—P. 19. "The effects of wine upon the system call for but little description. *They* resemble very closely those of spirit, but *it* appears to exhilarate the mind much more than the *latter*, before its intoxicating operation is manifested."—P. 195. Such faults, in some instances, have rendered the meaning obscure, if not unintelligible. The book, however, as a whole, is creditable to its authors, and will doubtless be acceptable and useful to many persons; and if, as will probably be the case (unless some other similar work should appear), this should come to a second edition, we are quite sure that its authors are capable of greatly improving it, and adapting it more completely to the purposes for which it is intended.

